# Measles seropositivity in previously vaccinated individuals: a systematic review and meta-analysis

**DOI:** 10.1016/j.eclinm.2025.103564

**Published:** 2025-11-03

**Authors:** Mohammed Mustafa, Alex S. Reznik, Juan Uribe, Joel Mintz, Sabrina Taldone, Bhavarth Shukla, Mohammed Raja, Yoichiro Natori

**Affiliations:** a Department of Internal Medicine, University of Miami, Miami, FL, USA; b Medical Scientist Training Programme, University of Miami Miller School of Medicine, Miami, FL, USA; c Division of Infectious Diseases, Department of Internal Medicine, University of Miami, Miami, FL, USA

**Keywords:** Measles, Vaccination, Waning, Antibody, Seropositivity, Immunisation, Immunity, Meta-analysis

## Abstract

**Background:**

With the resurgence of measles outbreaks globally it is important to understand how seropositivity induced by measles vaccination wanes over time. We aimed to measure the prevalence of measles seropositivity in vaccinated individuals and assess changes in antibody levels over time since vaccination.

**Methods:**

We conducted a systematic review and meta-analysis by searching PubMed, Embase, Scopus, Web of Science, and Cochrane Library databases from January 1980 to April 2025 for studies reporting serological outcomes following measles-containing vaccination. The search criteria included clinical trials, cross-sectional studies, cohort studies, and case series with ≥10 participants. The primary outcome was the proportion of vaccinated individuals seropositive for measles IgG antibodies. Meta-regression was performed to assess the relationship between seropositivity and time since vaccination. The study was registered with PROSPERO, CRD420251026672.

**Findings:**

The search identified 2348 unique records. Following title and abstract screening, 89 articles were selected for full-text review, of which 18 studies were included, encompassing 23,236 vaccinated individuals from 10 countries. Overall pooled proportion of seropositive individuals was 87·8% (95% Confidence Interval [CI] 83·9%–91·2%, I^2^ = 97·9%). Most individuals included in the analysis had received at least two doses of measles-containing vaccine, whereas evidence for single-dose recipients was limited. Single-dose recipients displayed a pooled seropositivity of 84·3% (95% CI, 71·6–93·9; 6 studies) and multi-dose recipients (individuals who received ≥2 doses) exhibited 88·8% (95% CI, 84·4–92·6; 15 studies; p = 0·252 for between-group heterogeneity). Notably, only one study included solely single-dose recipients, highlighting that estimates for this group are based on limited data. The overall seropositivity declined with increased time between vaccination and serological assessment: 92·9% (95% CI, 84·3%–98·4%) within 10 years, 87·0% (95% CI, 70·4%–97·4%) at 11–15 years, 82·8% (95% CI, 67·8%–93·9%) at 16–22 years, and 81·9% (95% CI, 30·8%–100%) at ≥23 years post-vaccination. Meta-regression suggested measles seropositivity declines at a rate of 0·48% per year in vaccinated individuals (β = −0·048, 95% CI, −0·078 to −0·018; p = 0·002).

**Interpretation:**

Ten years after measles vaccination, vaccinated individuals displayed high seropositivity. The observed decrease in measles seropositivity in the years after vaccination may indicate waning humoral immunity. However, this systematic review and meta-analysis was unable to assess the relationship between seropositivity and measles infection rates, thus further investigation is needed.

**Funding:**

No funding was received for the study design, data collection, analysis, interpretation, writing of the manuscript, or the decision to submit for publication.

**Research in context**

**Evidence before this study**

The prevalence of measles cases in vaccinated individuals and outbreaks in countries with high immunization rates suggests that waning seropositivity after vaccination may compromise population-level immunity. Although initial seroconversion after measles-mumps-rubella (MMR) vaccine is robust, the long-term durability of measles antibodies in vaccinated individuals is uncertain. Prior systematic reviews have primarily focused on vaccine safety and efficacy at single time points or exclusively in paediatric populations; however, no systematic reviews have examined measles seropositivity among vaccinated individuals across different time intervals following vaccination. The rate of antibody decline after measles vaccination remains unknown.

**Added value of this study**

Up to ten years after measles vaccination, overall seropositivity remains high in vaccinated individuals; however, the proportion of seropositive individuals declines over time. This study also identifies significant heterogeneity between assays for measles seropositivity. Neutralisation assays display higher seropositivity than chemiluminescent methods, demonstrating the need for uniform serological testing. To our knowledge, this is the first systematic review and meta-analysis to quantify waning measles seropositivity over time and comprehensively assess factors associated with the durability of measles seropositivity induced by vaccination.

**Implications of all the available evidence**

Measles seropositivity wanes after vaccination. Since measles IgG levels are secondary markers of immunity rather than direct correlates of clinical protection against measles, additional longitudinal studies examining the neutralizing capacity of measles antibodies, memory B cells, and T-cell responses are needed. Our study reveals crucial insights into the durability of measles antibodies and may help guide global measles elimination efforts. The documented reduction in measles seropositivity after vaccination demonstrates the potential value of targeted serological screening in adults vaccinated more than 20 years ago and the consideration of booster doses for groups at high risk of exposure. The significant differences in seropositivity by assay type emphasize the need for standardized serological surveillance using neutralisation-based methods. These findings support the urgent review of measles vaccination strategies, including cost-effectiveness comparisons between universal adult boosters versus risk-based or serologically-guided approaches, to durably achieve measles elimination without the risk of resurgence among previously protected cohorts.

## Introduction

Measles is among the most transmissible viral infections, with a basic reproduction number (R_0_) of 12–18, requiring population-level immunity between 92 and 95% to prevent sustained transmission.^1^, ^2^, ^3^ Prior to widespread vaccination, measles caused approximately 2·5 million deaths annually.^4^ Since 2000, measles vaccination has averted an estimated 60 million deaths worldwide.^5^ Despite these control measures, measles cases continue to resurge in countries with verified elimination and high vaccine coverage, including the USA and UK.^6^ Inadequate vaccine coverage is the primary driver of outbreaks, however incident measles outbreaks have been occasionally documented among fully vaccinated individuals, raising concerns about vaccine waning.^7^, ^8^, ^9^, ^10^, ^11^ These observations suggest that reduced seropositivity following measles vaccination may compromise population-level protection.^12^, ^13^, ^14^ However, individuals who experience secondary vaccine waning typically display a lower propensity for onward virus transmission and have milder clinical symptoms compared to unvaccinated individuals.^3^^,^^15^ The estimated frequency of secondary vaccine waning ranges between 2 and 10% among healthy vaccinated individuals^16^^,^^17^; however, whether this frequency differs between one-dose and two-dose recipients remains unknown. Several risk factors for reduced vaccine effectiveness have been identified, including host-related factors (such as immunosuppression and chronic disease) and vaccine-related factors (such as poor efficacy or incorrect administration).^17^ Nevertheless, previous research suggests that host-related factors, rather than vaccine-related factors, are the primary contributors to secondary vaccine waning.^17^

The measles-mumps-rubella (MMR) vaccine provides robust initial protection, with seroconversion rates of 93–95% after one dose and >99% after two doses.^18^^,^^19^ Cumulative evidence suggests measles antibodies decline over time following vaccination, particularly in single-dose recipients; however, the extent and public health ramifications of secondary vaccine waning are poorly characterized.^19^, ^20^, ^21^ Multiple studies report a decline in measles antibodies years after vaccination.^22^ The durability of antibodies differs between studies. Measles seropositivity in vaccinated populations ranges between 70% and 98% at comparable time points following vaccination.^19^^,^^21^^,^^22^ This heterogeneity likely reflects differences in study populations, vaccine formulations, and laboratory assays. Prior systematic reviews have evaluated vaccine efficacy at singular time points, primarily in paediatric populations.^23^, ^24^, ^25^ No comprehensive synthesis has quantified the rate of measles antibody decline. Hence, there remains a critical gap in characterising the long-term durability of measles seropositivity after vaccination.

Therefore, a systematic review and meta-analysis was conducted with three specific objectives: (1) quantify the prevalence of measles seropositivity among individuals with documented vaccination; (2) assess the rate of measles antibody decline over time since vaccination; and (3) identify factors associated with sustained seropositivity.

## Methods

This systematic review and meta-analysis was conducted in accordance with the PRISMA guidelines and the Cochrane Handbook for Systematic Reviews.^26^ The study was registered with PROSPERO, CRD420251026672.

### Search strategy and selection criteria

A comprehensive literature search was conducted on 11 April 2025, across five major databases: PubMed (MEDLINE), Embase, Scopus, Web of Science, and Cochrane Library for articles published in English between January 1980 and April 2025. Our search strategy was developed in consultation with a medical librarian and combined four main concept groups using Boolean operators: (1) measles-related terms, (2) vaccination status terms, (3) immunity waning indicators, and (4) population-based study terms. The core search included key terms such as “measles,” “seroprevalence,” “seropositivity,” “measles vaccine,” and “antibody”. Database-specific subject headings and controlled vocabulary were incorporated, including Medical Subject Headings (MeSH) terms in PubMed and Emtree terms in Embase. Complete search strategies for each database are provided in the appendix. The database search was supplemented by reviewing references of included studies and relevant reviews. Grey literature sources were not searched due to concerns about data quality and verification of vaccination status.

Studies were eligible if they reported the seropositivity, defined as the proportion of seropositive individuals previously vaccinated with at least one dose of measles-containing vaccine (MCV) at a defined time point who were one year of age or older at time of serological testing. Study-specific seropositivity thresholds were used due to variation in laboratory assays and cutoff values across studies and are described in Table 1. This approach maximizes inclusion but introduces heterogeneity that we addressed through subgroup analyses and meta-regression. Borderline or equivocal seropositivity results were classified as seropositive or seronegative based on individual study definitions.

**Table 1:** Characteristics of included studies reporting measles seropositivity after vaccination.

Author (Year)	Region	Study design	Population	Testing method	Assay cutoff	Study size	Seropositivity (%)	Quality assessment
Anichini (2020)	Italy	Cross-sectional	HCW	CLIA	>16·5 AU/mL	842	76·1	Moderate
Bianchi (2020)	Italy	Retrospective cohort	HCW	CLIA	>16·5 AU/mL	2000	89·1	High
Bianchi (2021)	Italy	Retrospective cohort	HCW	CLIA	>16·5 AU/mL	410	80·0	High
Castineras (2023)	Brazil	Cross-sectional	General	CLIA	>16·5 AU/mL	162	75·3	High
Chan (2024)	Hong Kong	Cross-sectional + Prospective cohort	General	PRNT	≥1:8	791	94·7	Moderate
Cohn (1994)	USA	Prospective cohort	General	FIAX	>12 IU/mL	1650	85·7	Moderate
Davidkin (2008)	Finland	Prospective cohort	General	ELISA	≥150 mIU/mL	183	95·3	Moderate
Dine (2004)	USA	Prospective cohort	General	PRNT	≥1:120	56	90·6	Moderate
Haralambieva (2011)	USA	Cross-sectional	Students	PRNT	≥210 mIU/mL	763	91·1	High
He (2013)	China	Cross-sectional	General	ELISA	≥200 mIU/mL	959	92·5	High
Kennedy (2019)	USA	Prospective cohort	General	PRMN	≥120 mIU/mL	90	97·8	Moderate
Kontio (2012)	Finland	Prospective cohort + Cross-sectional	General	ELISA	≥150 mIU/mL	119	86·6	Moderate
Kostinov (2021)	Russia	Cross-sectional	HCW + Pregnant	ELISA	≥0·18 IU	682	78·4	High
Kostinov (2021) (1)	Russia	Cross-sectional	HCW	ELISA	≥0·18 IU	1742	85·0	High
Leuridan (2010)	Belgium	Prospective cohort	Pregnant	ELISA	>300 mIU/mL	87	73·6	High
Nogareda (2020)	Mongolia	Cross-sectional	General	ELISA	≥150 mIU/mL	479	90·8	Moderate
Poethko-Müller (2011)	Germany	Cross-sectional	General	ELISA	≥350 mIU/mL	12 190	95·3	High
Vandermeulen (2007)	Belgium	Cross-sectional	Students	ELISA	≥350 IU/L	129	86·8	Moderate

HCW, healthcare workers; CLIA, chemiluminescent immunoassay; PRNT, plaque reduction neutralisation test; FIAX, fluorescent immunoassay; ELISA, enzyme-linked immunosorbent assay; PRMN, plaque microneutralisation assay.

Seropositivity cutoffs and assay platforms were extracted as reported by study authors.

Quality assessment was conducted using the Newcastle-Ottawa Scale (NOS) for non-randomised studies. Studies were classified as high quality if they scored greater than or equal to 7 points and moderate quality if they scored 5–6 points.

We included cross-sectional studies, prospective or retrospective cohort studies, and case series with greater than or equal to 10 participants. The following studies were excluded: (i) case reports or case series with less than 10 participants; (ii) studies without serological outcomes in vaccinated individuals; (iii) studies including only unvaccinated populations; (iv) modelling studies without serological outcomes; (v) outbreak vaccine-effectiveness studies lacking long-term follow-up; and (vi) studies including participants without vaccination status confirmed by medical records or self-report and (vii) population-level serosurveys, as they typically cannot definitively link seropositivity to vaccination versus natural infection.

Two reviewers (MM, AR) independently screened titles, abstracts, and full-text manuscripts, with conflicts resolved by a third reviewer (JU). Agreement between reviewers was assessed using Cohen's kappa statistic. All disagreements were resolved through discussion with a third reviewer (JU).

### Data extraction and quality assessment

Data extraction and synthesis was performed by two reviewers (MM, JU). Extracted variables included study characteristics (author, year of publication, country, design), participant demographics (age, gender, comorbidities), vaccination history (vaccine type, number of doses, age at vaccination, time since last dose), laboratory methods (assay type, seropositivity cutoff), and serological outcomes (proportion seropositive, geometric mean titres [GMTs] or concentrations [GMCs], with associated standard deviations or 95% confidence intervals [CIs]). Time since vaccination data was extracted from individual studies when available. Several studies (e.g., Anichini 2020) provided seropositivity data for multiple discrete time intervals, which were extracted separately. Individual patient data was not requested, and aggregate outcomes were extracted from publications.

Risk of bias for observational studies was assessed using the Newcastle-Ottawa Scale (NOS).^27^ Scores 7–9 were considered low risk of bias (high quality), 4–6 moderate, and ≤3 high risk.^28^ Two reviewers (MM, AR) independently completed the assessment and categorized risk of bias based on criteria related to selection, comparability, and outcome assessment. All disagreements were resolved through discussion with a third reviewer (JU). A sensitivity analysis limited to low-risk studies was performed.

### Data synthesis and analysis

The primary outcome of this study was overall measles seropositivity in subjects who had received at least one measles-containing vaccine (MCV) dose. Seropositivity was analyzed separately for recipients of one dose versus two doses. The secondary outcome was the change in measles seropositivity over time. For longitudinal analysis, subgroups were stratified by time since vaccination to assess for trends in seropositivity over time. The time intervals were prespecified based on available data distributions and logical time ranges to ensure sufficient data within each interval, including 0–10 years, 11–15 years, 16–22 years, and greater than or equal to 23 years.

Univariate and multivariate meta-regression were performed to explore potential sources of heterogeneity and evaluate associations between seropositivity and continuous variables, such as time since vaccination. The multivariate model included time since vaccination, assay type, vaccination dose group, population subgroup, and geographic region as moderators. For studies reporting seropositivity at multiple time points or in multiple subgroups, each measurement was included as a separate effect size in the meta-regression analysis, allowing all available data points to contribute to the estimation of time-dependent changes in seropositivity. A random-effects meta-analysis of seropositivity was conducted using restricted maximum likelihood (REML) estimation to account for between-study variance.^29^ Proportions were transformed using the Freeman-Tukey double arcsine transformation before pooling to stabilize variances and handle studies with proportions near 0% or 100%. Pooled estimates and their 95% CIs were calculated and back-transformed to the percentage scale for interpretability. Model fit was evaluated using R^2^ to assess the proportion of heterogeneity explained, and residual heterogeneity was tested using the Q statistic. Statistical heterogeneity was quantified using the I^2^ statistic, with thresholds of 25%, 50%, and 75% indicating low, moderate, and high heterogeneity, respectively.^30^ Studies reporting complete seropositivity were included using a continuity correction of 0·5 to enable variance calculations and avoid computational issues that arise when proportions equal exactly 1·0.

Subgroup analyses were conducted based on available data and aimed to explore sources of heterogeneity and identify factors associated with seropositivity. Data was stratified by time since last MCV dose, total dose count, gender, assay type, and geographic region. For gender-stratified analysis, four studies compared seropositivity between males and females within the same population. For vaccine dose analysis, reporting was heterogeneous: some studies reported data exclusively for single-dose recipients, others only for two-dose recipients, some stratified results by both dose groups, and several did not specify dose information. For two-dose recipients, time since vaccination was calculated from the second dose when specified, or from the last dose when dose-specific timing was not reported. Geographic regions were classified according to the six WHO regions (African Region, Region of the Americas, South-East Asia Region, European Region, Eastern Mediterranean Region, and Western Pacific Region). We included all population types in the primary analysis to maximize statistical power and generalizability, while conducting pre-specified subgroup analyses to explore heterogeneity. This approach balances inclusivity with the ability to identify population-specific differences. For population type analysis, all studies reported on a single population except Kostinov 2021 (1),^31^ which included healthcare workers and pregnant individuals with outcome data reported separately for each group; these were treated as independent data points for analysis.

Laboratory methods included enzyme-linked immunosorbent assay (ELISA), chemiluminescent immunoassay (CLIA), and plaque reduction neutralisation test (PRNT). A microneutralisation-based variant of PRNT, known as plaque reduction microneutralisation (PRMN), was included under the same analytic category due to its methodological and interpretative similarity to traditional neutralisation testing.

All analyses were conducted using R version 4·5·0 with the ‘meta’ package (version 8·1–0) (R Foundation for Statistical Computing, Vienna, Austria). Publication bias was assessed using funnel plots and Egger's regression test for asymmetry. A p-value <0·05 was considered statistically significant for all analyses. No protocol deviations occurred during the review process.

### Role of the funding source

No funding was received for the study design, data collection, analysis, interpretation, writing of the manuscript, or the decision to submit for publication.

## Results

### Study selection and characteristics

**Fig. 1:**
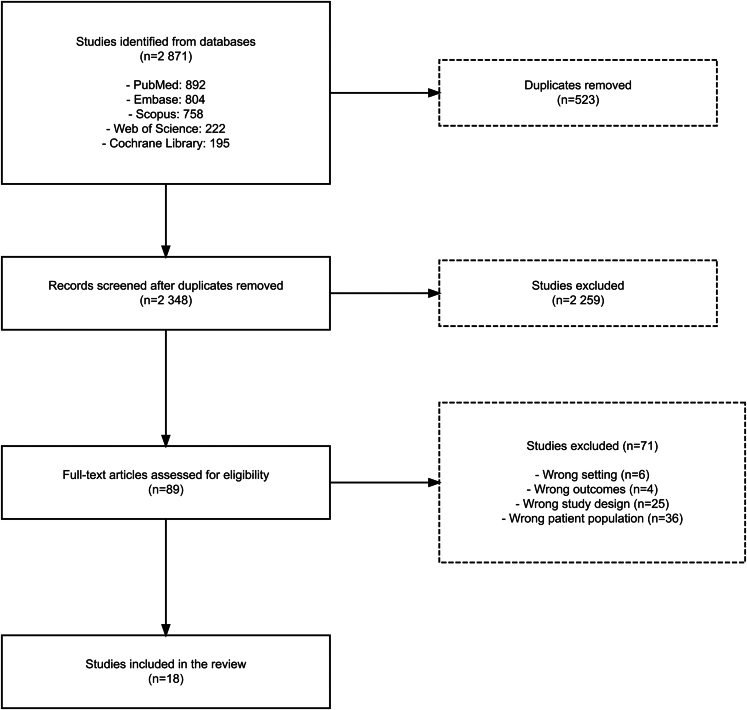
**PRISMA flow diagram of study selection**. A total of 2871 records were identified through database searches. After removing 523 duplicates, 2348 records were screened. Of these, 2259 were excluded during title and abstract screening. The remaining 89 full-text articles were assessed for eligibility, resulting in the exclusion of 71 studies for reasons including wrong setting, outcomes, study design, or population. Eighteen studies were ultimately included in the review.

The systematic literature search yielded 892 records from PubMed, 804 from Embase, 758 from Scopus, 222 from Web of Science, and 195 from Cochrane Library, resulting in 2348 records after duplicate removal. Following title and abstract screening, 89 articles were selected for full-text review of which 71 articles were excluded. Ultimately, 18 studies met inclusion criteria (Fig. 1).^21^^,^^31^, ^32^, ^33^, ^34^, ^35^, ^36^, ^37^, ^38^, ^39^, ^40^, ^41^, ^42^, ^43^, ^44^, ^45^, ^46^ Of note, the search criteria included clinical trials; however, none met inclusion criteria. For title and abstract screening (n = 2348), reviewers achieved substantial agreement (κ = 0·622, 90·5% agreement). For full-text review (n = 89), agreement was moderate (κ = 0·573, 83·5% agreement), reflecting the increased complexity of applying detailed eligibility criteria. The studies included in this systematic review and meta-analysis were published between 1994 and 2024 and encompassed 23 236 participants with documented vaccinations from 10 countries across North/South America, Europe, and Asia. The characteristics of included studies are summarized in Table 1. Study designs included cross-sectional (9 studies, 50·0%), prospective cohort (5 studies, 27·8%), retrospective cohort (2 studies, 11·1%) and two studies with a mixed prospective cohort and cross-sectional design (11·1%). Sample sizes ranged from 56 to 12,190 participants. Seven studies included both 1-dose and ≥2-dose MCV recipients, of which five provided results stratified by dose number. Ten studies reported exclusively on participants who had received ≥2 doses, while one study included only a 1-dose cohort.

**Appendix Fig. S1:**
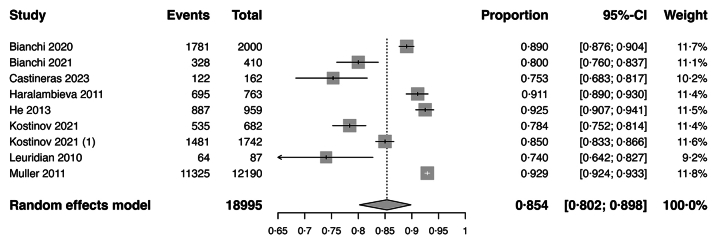
**Sensitivity analysis restricted to high-quality studies.** Random-effects meta-analysis of measles seropositivity limited to studies rated as high quality (Newcastle–Ottawa Scale score ≥7). The pooled seropositivity was 87·2% (95% CI, 81·1–92·2%) across 19,051 individuals. Heterogeneity remained high (I^2^ = 97·2%, τ^2^ = 0·017, p < 0·0001), indicating substantial between-study variability despite this restriction.

The most common serological assessment method was ELISA (9 studies, 50·0%), although individual cut-offs for seropositivity varied across studies. Overall study quality was moderate to high: NOS scores ranged from 5 to 8, with 9 studies (50·0%) rated as high quality (score greater than or equal to 7) and 9 moderate (score 4–6) (Appendix Table S1). No studies were excluded due to quality, but sensitivity analysis restricted to high-quality studies (NOS score ≥7) yielded similar results, with a pooled seropositivity of 85·4% (95% CI, 80·2–89·8%) (Appendix Fig. S1).

### Overall seropositivity

**Fig. 2:**
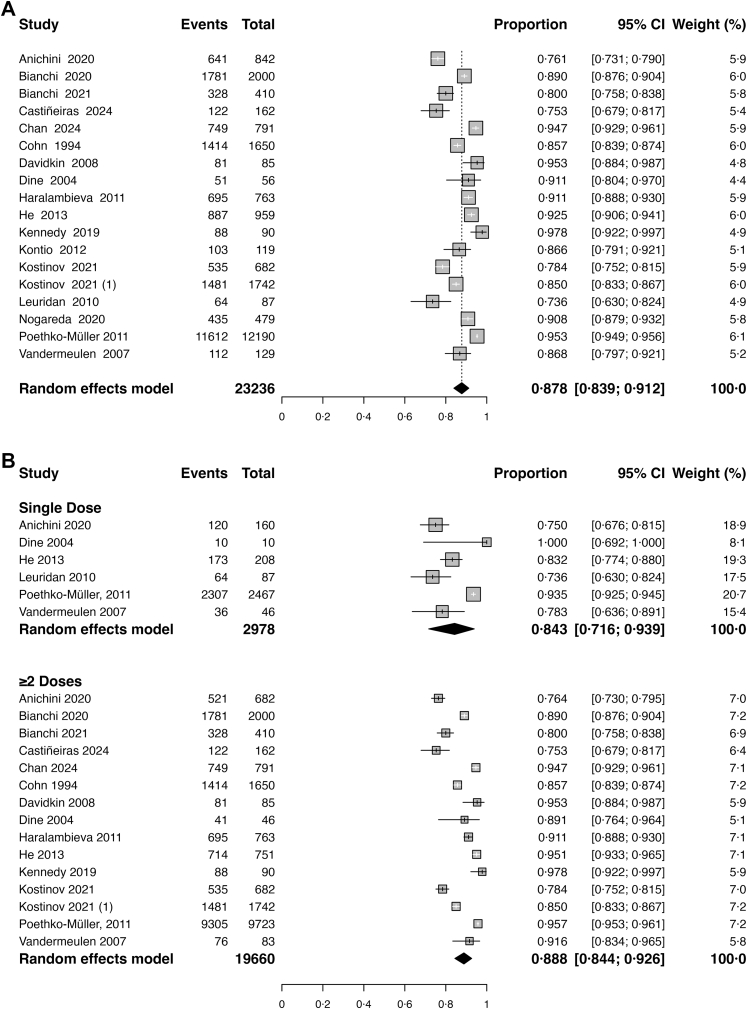
**a. Overall pooled measles seropositivity**. Random-effects meta-analysis of measles seropositivity proportions. Events represent the number of individuals who tested positive for measles antibodies (seropositive), while Total indicates the total number of individuals tested in each study. The pooled seropositivity was 87·8% (95% CI, 83·9–91·2%) across 23,236 individuals. Between-study heterogeneity was high (I^2^ = 96·8%), indicating substantial variability in seropositivity estimates across settings and populations. **b. Pooled measles seropositivity by vaccine dose**. Random-effects meta-analysis stratified by vaccination history. Events represent the number of individuals who tested positive for measles antibodies (seropositive), while Total indicates the total number of individuals tested in each study. Pooled seropositivity was 84·3% (95% CI, 71·6–93·9%) among single-dose recipients (n = 2978) and 88·8% (95% CI, 84·4–92·6%) among ≥2-dose recipients (n = 19,660). Despite higher early protection with ≥2 doses, long-term waning was observed in both groups. Heterogeneity remained high in both subgroups (I^2^ > 90%).

Pooled seropositivity was 87·8% (95% CI, 83·9%–91·2%) across 23,236 vaccinated individuals (Fig. 2a). There was substantial heterogeneity between studies (I^2^ = 97·9%, p < 0·001). When stratified by number of MCV doses, single-dose recipients exhibited a pooled seropositivity of 84·3% (95% CI, 71·6–93·9; 6 studies), compared with 88·8% (95% CI, 84·4–92·6; 15 studies) among those receiving ≥2 doses (p = 0·252 for between-group heterogeneity) (Fig. 2b).

### Waning seropositivity over time

**Fig. 3:**
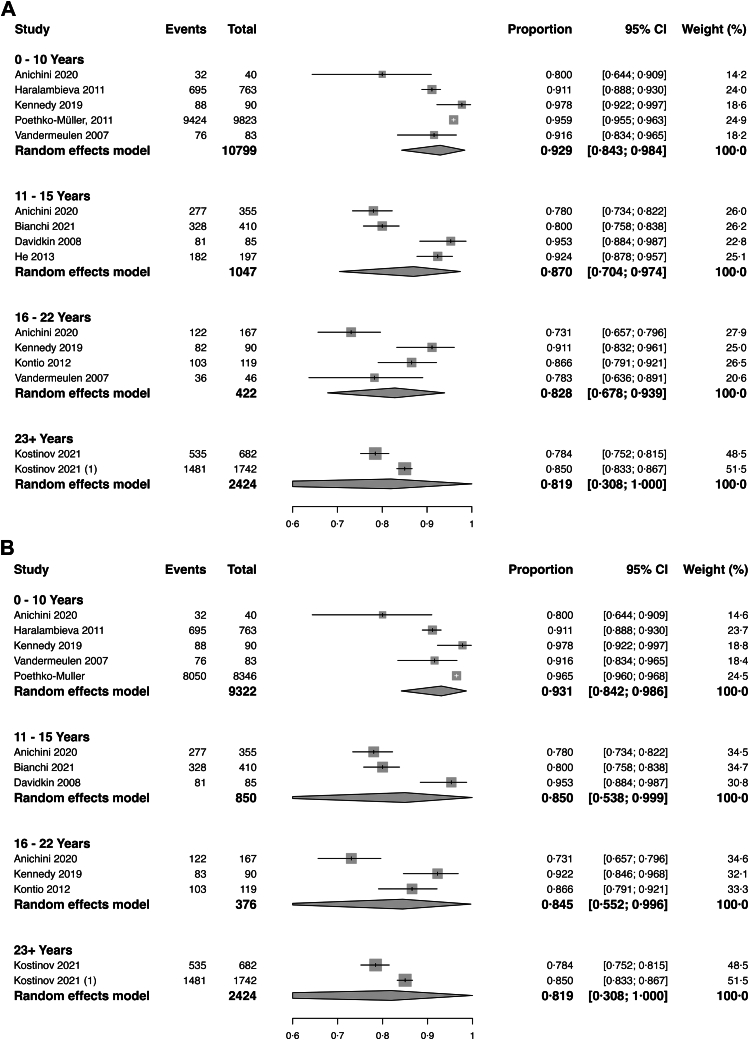
**a. Pooled measles seropositivity by time since vaccination**. Random-effects meta-analysis stratified by interval since vaccination. Seropositivity was highest within 0–10 years (92·9%, 95% CI, 84·3–98·4%; n = 10,799) and progressively declined across later intervals: 87·0% (70·4–97·4%; n = 1047) at 11–15 years, 82·8% (67·8–93·9%; n = 422) at 16–22 years, and 81·9% (30·8–100·0%; n = 2424) at ≥23 years. Substantial heterogeneity was observed across subgroups (I^2^ > 85%). **b. Pooled measles seropositivity by time since vaccination restricted to two-dose recipients**. Random-effects meta-analysis restricted to individuals who had received two vaccine doses. Seropositivity was highest within 0–10 years (93·1%, 95% CI, 84·2–98·6%; n = 9322) and declined thereafter: 85·0% (53·8–99·9%; n = 850) at 11–15 years, 84·5% (55·2–99·6%; n = 376) at 16–22 years, and 81·9% (30·8–100·0%; n = 2424) at ≥23 years. Results support a progressive decline in seropositivity over time, although wide confidence intervals reflect limited sample sizes in later strata.

**Fig. 4:**
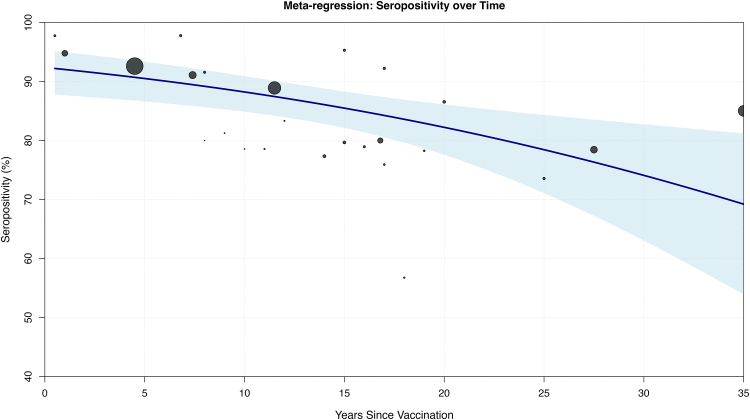
**Univariable meta-regression of measles seropositivity by time since vaccination**. A random-effects meta-regression model (REML estimator) was fitted to assess the association between years since vaccination and seropositivity. Each circle represents a time-specific seropositivity estimate, with marker size proportional to study weight. The fitted regression line (solid blue) and its 95% confidence interval (shaded region) demonstrate a significant decline in seropositivity with increasing time since vaccination (β = −0·039, p = 0·015). Residual heterogeneity remained high (I^2^ = 94·8%), with the model explaining 21·4% of between-study variability (R^2^).

Twelve studies reported data on seropositivity by time since vaccination. Three of these studies reported multiple data points, which were included individually for analysis. Seropositivity decreased with longer time elapsed since the last measles vaccine dose. For individuals assessed 0–10 years post-vaccination, the pooled seropositivity was 92·9% (95% CI, 84·3%–98·4%; I^2^ = 90·8%; 5 estimates). For those assessed 11–15 years post-vaccination, it was 87·0% (95% CI, 70·4%–97·4%; I^2^ = 91·9%; 4 estimates). For individuals 16–22 years post-vaccination, it was 82·8% (95% CI, 67·8–93·9%; I^2^ = 81·3%; 4 estimates), and for those with follow-up greater than or equal to 23 years it was 81·9% (95% CI, 30·8%–100%; I^2^ = 93·0%; 2 estimates) (Fig. 3a). When limited to individuals who received ≥2 MCV doses (13 data points; 12,972 observations), there was a similar trend in seropositivity over time: 93·1% (95% CI, 84·2–98·6; I^2^ = 92·4%) at 0–10 years (5 estimates), 85·0% (95% CI, 53·8–99·9; I^2^ = 89·5%) at 11–15 years (3 estimates), 84·5% (95% CI, 55·2–99·6; I^2^ = 88·5%) at 16–22 years (3 estimates), and 81·9% (95% CI, 30·8–100; I^2^ = 93·0%) at ≥23 years (2 estimates) (Fig. 3b). Meta-regression demonstrated no evidence of difference in the trajectory of waning between analyses including all doses versus those limited to ≥2 doses (p for interaction = 1·00 across all time strata) (Fig. 4).

A univariate mixed-effects meta-regression was performed to examine the relationship between time since vaccination and seropositivity across 25 time points spanning 0·5–35 years of follow-up. The analysis revealed a significant negative association between years since vaccination and seropositivity (β = −0·048, 95% CI, −0·078 to −0·018, p = 0·002). This model accounted for 34·7% of the between study heterogeneity (R^2^ = 34·7%). However, residual heterogeneity remained (τ^2^ = 0·280, I^2^ = 90·3%), with the test for residual heterogeneity being highly significant (QE = 167·68, df = 23, p < 0·001). The intercept of 2·50 (95% CI, 1·98–3·01, p < 0·001) corresponds to an estimated seropositivity of 92·4% immediately following vaccination.

A multivariate meta-regression including assay type, vaccine doses, population type, and geographic region as additional covariables explained 21·4% of between-study heterogeneity (R^2^ = 21·4%). Time since vaccination remained the strongest predictor (β = −0·049; OR 0·95; 95% CI, 0·93–0·98; p < 0·001). Neutralisation assays yielded slightly higher seropositivity estimates than ELISA or CLIA, while students showed lower seropositivity compared with the general population. Most residual heterogeneity originated between studies (ICC = 87%), underscoring unmeasured contextual factors. Full model outputs, sensitivity analyses, and subgroup predictions are presented in the Supplement.

### Subgroup analyses

#### Gender

Four studies included gender-stratified seropositivity data with a total of 7087 males and 7727 females. There was no significant difference in pooled seropositivity between genders. Seropositivity was 89·9% (95% CI, 89·2%–90·6%; I^2^ = 96·7%) and 89·7% (95% CI, 89·0%–90·4%; I^2^ = 62·5%) in females and males, respectively (p = 0·69 for heterogeneity between genders) (Appendix Table S2).

#### Assay type

The majority of studies (9; 50%) used ELISA to determine seropositivity, with a pooled seropositivity of 91·3% (95% CI, 90·9%–91·7%; I^2^ = 97·2%). Four studies (22·2%) used CLIA with a pooled seropositivity of 84·2% (95% CI, 83·0%–85·4%; I^2^ = 96·6%), four studies (22·2%) used a neutralisation method (PRNT/PRMN) with a pooled seropositivity of 93·2% (95% CI, 92·0%–94·4%; I^2^ = 71·3%), and one study (5·6%) used FIAX with a reported seropositivity of 85·7% (95% CI, 84·0%–87·4%) (p < 0·001 for overall differences by assay method; Appendix Table S2). Table 1 describes assay cutoffs used by each study.

#### Region

Seropositivity also varied significantly by region. Studies from Asia (3; 16·7%) reported the largest seropositivity of 93·6% (95% CI, 92·6%–94·6%; I^2^ = 44·3%). Europe had the largest number of studies (10; 55·6%) and reported a pooled seropositivity of 89·9% (95% CI, 89·5%–90·3%; I^2^ = 98·1%). Studies performed in the Americas (5; 27·8%) were determined to have a pooled seropositivity of 87·1% (95% CI, 85·8%–88·4%; I^2^ = 90·0%) (p < 0·001 across regions; Appendix Table S2).

#### Population

Meta-analysis of seropositivity by population type revealed substantial variation between groups. The pooled seropositivity was highest in the general population at 92·2% (95% CI, 89·9%–94·0%; I^2^ = 92·5%), followed by 86·5% in healthcare workers (95% CI, 85·4%–87·6%; I^2^ = 92·0%), 78·3% in students (95% CI, 76·1%–80·5%; I^2^ = 76·0%), and 78·0% in pregnant individuals (95% CI, 74·9%–81·1%; I^2^ = 11·0%) (Appendix Table S2).

Heterogeneity was considerable overall (I^2^ = 96·6%) and within most subgroups, indicating significant between-study variability. A test for subgroup differences showed a statistically significant effect (Q = 25·02, df = 3, p < 0·001).

### Publication bias

**Appendix Fig. S2:**
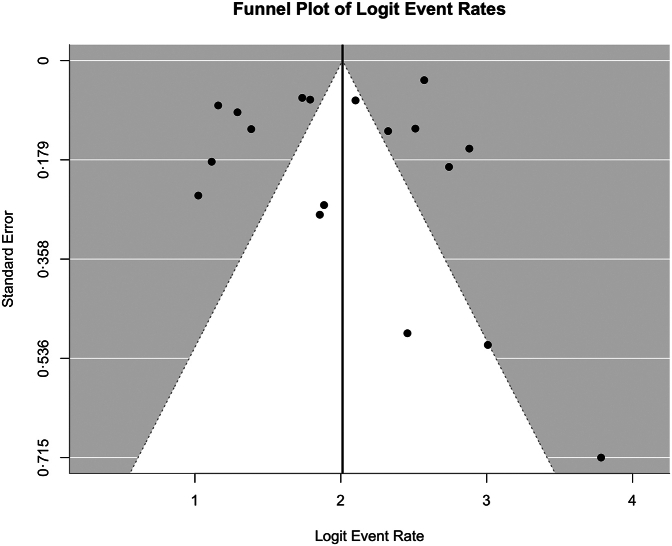
**Funnel plot assessing publication bias.** Funnel plot of studies reporting overall measles seropositivity. Effect sizes (logit-transformed proportions) are plotted against their standard errors. The funnel plot demonstrated reasonable symmetry around the pooled effect, with studies distributed evenly on both sides of the summary estimate.

Assessment of publication bias using Egger's regression test (p = 0·266) and Begg's rank correlation test (p = 0·201) showed no significant funnel plot asymmetry for overall seropositivity, suggesting the absence of publication bias. The funnel plot demonstrated reasonable symmetry around the pooled effect, with studies distributed evenly on both sides of the summary estimate (Appendix Fig. S2).

## Discussion

In this systematic review and meta-analysis, we found that vaccinated individuals maintain high seropositivity one decade after measles vaccination. We also demonstrate that measles seropositivity in vaccinated individuals declines over time. Our data identifies 12·2% of vaccinated individuals lack detectable measles antibodies, rising to 18·1% after two decades post-vaccination. These findings suggest routine childhood MCV vaccination may not grant lifelong immunity and raises questions about whether additional vaccination strategies might be worth investigating in the future. While serosurvey data from the 2009–2010 U.S. NHANES study reported high overall measles seroprevalence, it did not stratify participants by vaccination status, limiting conclusions about vaccine-induced immunity specifically.^47^ Notably, the study observed a decline in seropositivity from children aged 6–11 years (96·4%) to adults aged 30–39 years (88·6%), followed by an increase in older age groups, likely reflecting natural infection prior to widespread vaccination implementation in 1966. In addition, a measles epidemiological assessment conducted in Italy between 2013 and 2022 estimated that 9·2% of the Italian population in 2025 was susceptible to measles infection and this immunity gap was particularly high in individuals born in the 1980s and 1990s.^48^ These patterns support our interpretation that seropositivity derived from measles vaccination may wane over time.

The temporal decline in seropositivity–from 92·9% within 10 years to 81·9% after 23 years–which highlights the need for further research on translation of seropositivity to immunity in populations. Our meta-regression quantifies this decline at 0·48% annually, which estimates 5 per 1000 vaccinated individuals may become seronegative per year.

The difference in seropositivity between single-dose (84·3%) and two-dose recipients (88·8%) was statistically insignificant and smaller than anticipated, as initial measles vaccine trials and early surveillance studies demonstrated second doses typically increase seroconversion rates by 5–7%.^15^ Although two MCV doses provided higher early seropositivity, our analyses demonstrate that long-term waning occurs irrespective of dose number. Importantly, only one study exclusively evaluated single-dose recipients, while most studies included participants with ≥2 doses, limiting the strength of conclusions for single-dose individuals. Restriction to ≥2-dose recipients yielded estimates nearly identical to the full cohort, with no significant difference in the trajectory of decline across time strata. These findings suggest that while two doses confer stronger initial seropositivity, additional strategies may be needed to sustain long-term seropositivity induced by measles vaccination. This is particularly relevant since many countries transitioned to two-dose vaccination schedules specifically to address vaccine waning, without anticipating that antibodies would wane substantially regardless of initial dose number.^49^ However, it is important to recognize that seropositivity may not correlate with protective immunity against measles and no definitive conclusions ought to be drawn from seropositivity alone.

Assay methodology significantly influenced seropositivity measurements, with neutralisation assays reporting 93·2% seropositivity compared to 84·2% for CLIA. This disparity in seropositivity detection highlights the critical need for standardized serological testing in surveillance programmes. Among studies using gold-standard neutralization assays (PRNT or PMRN) with a threshold of ≥120 mIU/mL, seropositivity ranged from 90·6% to 97·8%, which was generally higher than in studies using commercial ELISA or CLIA platforms, where seropositivity varied more widely (76·1%–95·3%) and often used higher or inconsistent cutoffs.

These results confirm neutralisation assays to be the gold standard, though ELISA (91·3% seropositivity) remains acceptable for large-scale screening if properly calibrated against international standards or deployed in combination with confirmatory neutralisation assays.

While our inclusion criteria did not exclude younger participants, the identified studies predominantly focused on adolescents and adults, with limited data on children under 10 years. This reflects the research focus on long-term immunity rather than early post-vaccination responses. Our findings have several implications for measles control strategies. First, targeted serological screening could be considered for high-risk groups, including healthcare workers, international travelers, and adults born during periods of suboptimal vaccine coverage. Second, research is needed to evaluate the cost-effectiveness and optimal timing of booster doses in adult populations. Finally, mathematical modeling studies have demonstrated that minor waning of vaccine-induced antibodies can influence measles control in settings with high vaccine coverage.^13^ Our synthesis of long-term seropositivity data after vaccination provides information for future modeling studies, which could help inform evidence-based policy decisions.

This meta-analysis is the largest synthesis of measles vaccine-induced seropositivity duration to date, including over 23,000 individuals across six continents. Nevertheless, several limitations merit consideration. First, measles-specific IgG antibodies are an imperfect surrogate for clinical protection. While IgG levels correlate with immunity at the population level and remain the most practical biomarker for large-scale assessment,^50^^,^^51^ individuals with undetectable antibodies may still be protected through cellular immunity or anamnestic responses.^52^ Conversely, some individuals with detectable antibodies may not be fully protected. This fundamental limitation means our findings reflect antibody persistence rather than true protective immunity, potentially overestimating the population at risk. Second, the cross-sectional design of most included studies prevents direct observation of antibody kinetics within individuals. Third, heterogeneous reporting of vaccination details limited our ability to assess the influence of factors such as age at vaccination or vaccine formulation on durability of antibody levels. The use of variable seropositivity thresholds across studies represents a significant limitation, as these thresholds may not be directly comparable across different assay platforms. Fourth, a small number of the included studies reported results using international standards for protective antibody levels, preventing comprehensive assessment of the proportion of individuals below established correlates of protection. Four studies followed WHO international standards (PRN titre ≥120) and were analysed separately (Appendix Table S2).^53^ Finally, our exclusion of grey literature may have introduced publication bias, though our statistical assessment did not find evidence of small-study effects.

Measles seropositivity declines over time; however, seropositivity rates remain high even a decade after vaccination. Future studies are needed to determine the clinical significance of reduced measles seropositivity in vaccinated individuals for both individual and population-level immunity against measles infection.

## Contributors

MM, AR, ST, MR, BS, and YN conceived the study and designed the protocol. MM, AR, JU, and JM undertook the literature search and curated the data. MM, AR, JU, JM, MR, BS, and YN developed the methodology and protocol. MM, JU, and YN conducted the statistical analysis and prepared the visualisations. MM and JU accessed and verified the underlying data. ST, BS, MR, and YN provided supervision and mentorship. MM drafted the protocol and the final manuscript, with AR contributing sections; all authors contributed text and provided critical revisions. YN served as the senior author and guarantor of the review. All authors had full access to the data, were accountable for the accuracy and integrity of the work and approved the final version of the manuscript.

## Data sharing statement

All data used in this study was obtained from publicly available sources. Code for data processing used for analysis is available upon request.

## Declaration of interests

AR was supported by NIH training grant #5T32GM145462-03. BS received an internal grant award from the University of Miami for an unrelated project (UM AWD-VIR TSA-01). YN received a grant from Eurofins Viracor™ for unrelated research, has received consulting fees from Nobel Pharma™ and Kamada™, has received honoraria for lectures from Eurofins Viracor™ and Kamada™, and is an executive committee member for the American Society of Transplantation and the International Society of Heart and Lung Transplantation.
