# Supplementary material for: Measles seropositivity in previously vaccinated individuals: a systematic review and meta-analysis
Source: eClinicalMedicine. 2025 Nov 3;89:103564. doi: 10.1016/j.eclinm.2025.103564 (PMC12675032; doi:10.1016/j.eclinm.2025.103564)
Supplement: Appendix Table S1 [file mmc1.docx]

**Appendix Table 1. Quality assessment of included studies using the Newcastle–Ottawa Scale (NOS)**

| **Author (Year)** | **Selection (max 4)** | **Comparability (max 2)** | **Outcome (max 3)** | **Total Score (max 9)** | **Quality Assessment** |
| --- | --- | --- | --- | --- | --- |
| **Anichini 2020** | 3 | 0 | 2 | 5 | Moderate |
| **Bianchi 2020** | 3 | 2 | 3 | 8 | High |
| **Bianchi 2021** | 3 | 2 | 3 | 8 | High |
| **Castineras 2023** | 3 | 2 | 2 | 7 | High |
| **Chan 2024** | 2 | 1 | 3 | 6 | Moderate |
| **Cohn 1994** | 3 | 0 | 2 | 5 | Moderate |
| **Davidkin 2008** | 3 | 0 | 2 | 5 | Moderate |
| **Dine 2004** | 3 | 0 | 2 | 5 | Moderate |
| **Haralambieva 2011** | 3 | 1 | 3 | 7 | High |
| **He 2013** | 3 | 1 | 3 | 7 | High |
| **Kennedy 2019** | 2 | 1 | 3 | 6 | Moderate |
| **Kontio 2012** | 2 | 1 | 3 | 6 | Moderate |
| **Kostinov 2021** | 3 | 1 | 3 | 7 | High |
| **Kostinov 2021 (1)** | 3 | 1 | 3 | 7 | High |
| **Leuridan 2010** | 4 | 1 | 2 | 7 | High |
| **Poethko-Müller 2011** | 3 | 1 | 3 | 7 | High |
| **Nogareda 2020** | 2 | 1 | 3 | 6 | Moderate |
| **Vandermeulen 2007** | 3 | 0 | 3 | 6 | Moderate |

The Newcastle–Ottawa Scale (NOS) evaluates the methodological quality of non-randomised studies across three domains: Selection (maximum 4 points), Comparability (maximum 2 points), and Outcome assessment (maximum 3 points), for a total maximum score of 9.

Studies were rated as high quality if they scored greater than or equal to 7 points and as moderate quality if they scored 4–6 points. No studies scored below 4. Scoring was conducted independently by two reviewers, with discrepancies resolved by a third reviewer.
