# Supplementary material for: Measles seropositivity in previously vaccinated individuals: a systematic review and meta-analysis
Source: eClinicalMedicine. 2025 Nov 3;89:103564. doi: 10.1016/j.eclinm.2025.103564 (PMC12675032; doi:10.1016/j.eclinm.2025.103564)
Supplement: Appendix Table S2 [file mmc2.docx]

**Appendix Table 2. Subgroup analysis of pooled measles seropositivity by study and methodological characteristics**

| **Subgroup** | **No. of Studies** | **Total Sample  Size** | **Weighted Seropositivity (%)** | **95% CI** | **p-value^†^** |
| --- | --- | --- | --- | --- | --- |
| ***Gender*** |  |  |  |  | p = 0·69 |
| Female | 3 | 7 727 | 89·9 | 89·2–90·6 |  |
| Male | 3 | 7 087 | 89·7 | 89·0–90·4 |  |
| ***Assay Type*** |  |  |  |  | **p < 0**·**001** |
| CLIA | 4 | 3 414 | 84·2 | 83·0–85·4 |  |
| ELISA | 9 | 16 472 | 91·3 | 90·9–91·7 |  |
| FIAX | 1 | 1 650 | 85·7 | 84·0–87·4 |  |
| NEUT | 4 | 1 700 | 93·2 | 92·0–94·4 |  |
| ***Geographic Region*** |  |  |  |  | **p < 0**·**001** |
| Americas | 5 | 2 721 | 87·1 | 85·8–88·4 |  |
| Asia | 3 | 2 229 | 93·6 | 92·6–94·6 |  |
| Europe | 10 | 18 286 | 89·9 | 89·5–90·3 |  |
| ***Population Type***** |  |  |  |  | **p < 0**·**001** |
| General | 10 | 17 182 | 92·2 | 91·8–92·6 |  |
| HCW | 4 | 3 992 | 86·5 | 86·4–87·6 |  |
| Pregnant Individuals | 3 | 681 | 78·0 | 74·9–81·1 |  |
| Students | 2 | 1 381 | 78·3 | 76·1–80·5 |  |

CI = confidence interval; MCV = measles-containing vaccine; CLIA = chemiluminescent immunoassay; ELISA = enzyme-linked immunosorbent assay; FIAX = fluorescent immunoassay; NEUT = neutralisation-based assay (includes PRMN and PRNT methods, grouped due to methodological similarity); HCW = healthcare workers.

^†^p-values represent tests for between-group differences in pooled seropositivity using random-effects meta-analysis with Freeman–Tukey transformation, REML heterogeneity estimation, and Hartung–Knapp adjustment.

*Some studies contributed data to both the 1-dose and ≥2-dose categories; others reported overall seropositivity without dose stratification and were excluded from this subgrouping.

**Kostinov 2021 (1) contributed data for two distinct populations (healthcare workers and pregnant individuals), which were analysed separately.
