# Supplementary material for: Measles seropositivity in previously vaccinated individuals: a systematic review and meta-analysis
Source: eClinicalMedicine. 2025 Nov 3;89:103564. doi: 10.1016/j.eclinm.2025.103564 (PMC12675032; doi:10.1016/j.eclinm.2025.103564)
Supplement: Supplementary Appendix [file mmc3.docx]

**Supplementary Appendix**

**Multivariate Meta-Regression**

In multivariable meta-regression, time since vaccination was consistently associated with lower odds of measles seropositivity across all model specifications. In the nested random-effects model, which partitioned variance at both study and observation levels, each additional year post-vaccination was associated with a 4·9% relative reduction in the odds of being seropositive (β = –0·049; OR 0·95, 95% CI 0·93–0·98; p = 0·0012). This decline remained robust after applying cluster-robust standard errors (p = 0·033).

Assay type did not significantly influence results, although neutralisation-based assays tended to report higher seropositivity compared with ELISA, while CLIA assays yielded lower estimates. In predicted margins, average seropositivity was 91·3% for neutralisation assays, 87·5% for ELISA, and 83·7% for CLIA.

Population subgroup analyses suggested differences in immunity. Students had significantly lower seropositivity than the general population (β = –0·69; OR 0·50, 95% CI 0·23–1·10; p = 0·084), whereas health-care workers (HCWs) and pregnant women showed numerically lower but non-significant differences. Predicted seropositivity was highest among the general population (92·3%) and lower among students (84·4%), HCWs (80·4%), and pregnant women (80·6%).

Between-study variance accounted for most of the residual heterogeneity (τ² = 0·138 at the study level vs 0·021 within studies), with an intraclass correlation of 0·87, indicating that nearly 87% of unexplained variability lay between rather than within studies. Models adjusting for vaccine dose did not identify a statistically significant effect of receiving one versus two doses, but overall trends were consistent with higher early seropositivity after two doses followed by parallel waning over time.

**Study Quality**

In sensitivity analyses limited to high-quality studies (NOS ≥7; nine studies), the pooled estimate of measles seropositivity was 85·4% (95% CI, 80·2–89·8%). This value closely paralleled the overall meta-analysis, indicating that exclusion of lower-scoring studies did not materially alter the results. Nonetheless, heterogeneity remained considerable (I² = 97·4%), suggesting that the variability observed between studies is unlikely to be explained solely by study quality. Instead, factors such as regional vaccine program differences, assay variability, or unmeasured population characteristics may contribute to residual heterogeneity. These findings reinforce the robustness of the primary analysis while highlighting the continued need for methodological harmonization in future seroprevalence research.

**Comprehensive Search Strategy** (Search Date: April 11, 2025)

**PubMed** (MEDLINE via NCBI)

Filter(s): English; Human; 1/1/1980 - 4/1/2025

Total Results: 892

Search Strategy: (("Measles"[Mesh] OR measles[Text Word]) AND ("Seroprevalence"[Text Word] OR seropositivity[Text Word] OR antibody*[Text Word] OR "Immunoglobulin G"[Mesh] OR IgG[Text Word])) AND ("Measles-Mumps-Rubella Vaccine"[Mesh] OR "Measles Vaccine"[Mesh] OR measles vaccine*[Text Word] OR MMR vaccine*[Text Word] OR measles-mumps-rubella vaccine*[Text Word]) AND(vaccinated[Text Word] OR vaccination[Text Word] OR immunized[Text Word] OR immunisation[Text Word] OR post-vaccination[Text Word]) NOT (mumps[Title] OR rubella[Title])

**Embase**

Filter(s): English; Human; 1/1/1980 - 4/1/2025

Total Results: 804

Search Strategy: (('measles'/exp OR measles:ti,ab) AND ('seroprevalence'/exp OR seroprevalence:ti,ab OR seropositivity:ti,ab OR 'seroepidemiologic study'/exp OR seroepidemiology:ti,ab OR screening:ti,ab OR 'population based':ti,ab OR prevalence:ti,ab) AND ('measles vaccine'/exp OR 'measles mumps rubella vaccine'/exp OR 'measles vaccine':ti,ab OR 'mmr vaccine':ti,ab OR 'measles-mumps-rubella vaccine':ti,ab) AND (vaccinated:ti,ab OR vaccination:ti,ab OR immunized:ti,ab OR immunised:ti,ab OR 'post-vaccination':ti,ab)) NOT (mumps:ti OR rubella:ti OR genetic*:ti OR association*:ti OR transcriptom*:ti OR irradiat*:ti OR "B cell":ti OR "T cell":ti OR "memory B":ti OR polymorphism*:ti OR variant*:ti OR cytokine*:ti)

**Scopus**

Filter(s): English; Human; 1/1/1980 - 4/1/2025

Total Results: 758

Search Strategy: ( TITLE-ABS-KEY ( "measles antibody" ) OR TITLE-ABS-KEY ( "measles IgG" ) OR TITLE-ABS-KEY ( "measles immunoglobulin G" ) OR TITLE-ABS-KEY ( "measles immunity" ) OR TITLE-ABS-KEY ( measles AND seroprevalence ) OR TITLE-ABS-KEY ( measles AND seropositivity ) OR TITLE-ABS-KEY ( measles AND "seroepidemiologic study" ) OR TITLE-ABS-KEY ( measles AND seroepidemiology ) OR TITLE-ABS-KEY ( measles AND "population-based study" ) OR TITLE-ABS-KEY ( measles AND "screening study" ) OR TITLE-ABS-KEY ( measles AND "IgG titer" ) OR TITLE-ABS-KEY ( measles AND "IgG titre" ) ) AND TITLE-ABS-KEY ( "measles vaccine" OR "measles-mumps-rubella vaccine" OR "mmr vaccine" ) AND TITLE-ABS-KEY ( vaccinated OR vaccination OR immunized OR immunised OR "post-vaccination" ) AND NOT TITLE ( mumps OR rubella ) AND NOT TITLE-ABS-KEY ( genetic* OR association* OR irradiat* OR "B cell" OR "T cell" OR "memory B" OR polymorphism* OR variant* OR cytokine* ) AND PUBYEAR > 1979 AND PUBYEAR < 2026 AND ( LIMIT-TO ( DOCTYPE , "ar" ) ) AND ( LIMIT-TO ( EXACTKEYWORD , "Human" ) ) AND ( LIMIT-TO ( LANGUAGE , "English" ) )

**Web of Science**

Filter(s): English; Human; 1/1/1980 - 4/1/2025

Total Results: 222

Search Strategy: ((TS=("measles antibody") OR TS=("measles IgG") OR TS=("measles immunoglobulin G") OR TS=("measles immunity") OR TS=(measles AND seroprevalence) OR TS=(measles AND seropositivity) OR TS=(measles AND "seroepidemiologic study") OR TS=(measles AND seroepidemiology) OR TS=(measles AND "population-based study") OR TS=(measles AND "screening study") OR TS=(measles AND "IgG titer") OR TS=(measles AND "IgG titre")) AND TS=("measles vaccine" OR "measles-mumps-rubella vaccine" OR "mmr vaccine") AND TS=(vaccinated OR vaccination OR immunized OR immunised OR "post-vaccination”)) NOT (TI=(mumps OR rubella) OR TS=(genetic* OR association* OR transcription* OR irradiat* OR "B cell" OR "T cell" OR "memory B" OR polymorphism* OR variant* OR cytokine*))

**Cochrane**

Filter(s): English; Human; 1/1/1980 - 4/1/2025

Total Results: 195

Search Strategy:
MeSH descriptor: [Measles] explode all trees

MeSH descriptor: [Measles Vaccine] explode all trees

MeSH descriptor: [Measles-Mumps-Rubella Vaccine] explode all trees

MeSH descriptor: [Serologic Tests] explode all trees

(measles OR "measles vaccine" OR "measles mumps rubella vaccine" OR "mmr vaccine") AND (seroprevalence OR seropositivity OR seroepidemiologic study OR seroepidemiology OR "population based study" OR "screening study" OR "measles antibody" OR "measles IgG" OR "measles immunoglobulin G" OR "measles immunity" OR "IgG titer" OR "IgG titre") AND (vaccinated OR vaccination OR immunized OR immunised OR "post-vaccination") NOT (mumps OR rubella OR genetic* OR association* OR transcriptom* OR irradiat* OR "B cell" OR "T cell" OR "memory B" OR polymorphism* OR variant* OR cytokine*)

Additional Review: We reviewed reference lists of all included studies to identify additional relevant publications not captured in the electronic database searches. We also used Google Scholar to identify studies citing key included papers, particularly seminal works on measles immunity and vaccination. Finally, we reviewed reference lists of relevant systematic reviews and meta-analyses identified during the search process.
